# Midwifery Education Institutions in Italy Creation and Validation of Clinical Preceptors’ Assessment Tool: Students’ and Expert Midwives’ Views

**DOI:** 10.3390/nursrep10020021

**Published:** 2020-12-16

**Authors:** Paola Agnese Mauri, Ivan Cortinovis, Norma Nilde Guerrini Contini, Marta Soldi

**Affiliations:** 1 Department of Clinical Sciences and Community Health, Università degli Studi di Milan, via Manfredo Fanti 6, 20122 Milan, Italy; ivan.cortinovis@unimi.it; 2 Unit of Mother Child and Newborn Health, Fondazione IRCCS Ca’ Granda Ospedale Maggiore Policlinico, via Manfredo Fanti 6, 20122 Milan, Italy; norma.guerrini@gmail.com (N.N.G.C.); marta.soldi@gmail.com (M.S.)

**Keywords:** midwifery, nursing, clinical preceptor

## Abstract

*Background:* The aim of the study is to create and validate a midwifery preceptor’s evaluation form to be used by midwifery students. The International Confederation of Midwives recommends that clinical placements need to be supervised by a preceptor in order to be efficient for students who, in this way, gain competence and proper practice within the midwifery practical area. *Methods*: This is an observational multi-center transversal study and leads to the validation of an evaluation questionnaire. Methodically, the following steps were followed: literature review, focus group with midwifery students, meeting between expert midwives, creation of the preceptor’s assessment form, filling in of the forms by midwifery students and expert midwives, and validation of the form. The study was carried out in eight Italian universities and included eighty-eight midwifery students and eight midwives. *Results and Conclusion*: A midwifery preceptor’s assessment questionnaire was created made up of four attribute areas which, as a total, included 33 items. Cronbach’s alpha score was calculated after examining the forms filled in by students and expert midwives. An alpha score of 0.97–0.85 was obtained. The result was Pearson Correlation Coefficient *r* = 0.78.

## 1. Introduction

In Italy, the midwifery university’s major lasts three years, in which students need to achieve 180 university credits in order to graduate; 112 of them are for theoretical activities, and 68 are for clinical placements. One credit of theoretical activity corresponds to 15 h of classes and 10 h of individual study, and one credit of practical activity is the equivalent of 30 h of clinical placement (Ministerial Decree n. 70, 2004) [1]. Midwifery students have to complete 2040 h of supervised clinical placements. The health professional allocated to the student during clinical practice is the “facilitator of the training path, who orientates the student towards professional operation, demonstrating models linked to professional membership profile” [2]. In Italy specific training to be a clinical midwife preceptor or nurse preceptor is not required.

International literature both identifies “preceptor” [3,4] and “mentor” [5,6,7] as terms indicating an experienced midwife engaged in the practice of midwifery who is competent and willing to teach, observe and assess midwifery students during their practical/clinical learning [4]. In this study we chose to use the term preceptor to indicate the person allocated to students during their clinical placements.

## 2. Background

Clinical placements are fundamental to midwifery students [3,7,8] and the relationship between the student and the preceptor influences the student’s learning and the integration of theoretical information and practice [9,10]. The Nursing and Midwifery Council [11], the International Confederation of Midwives [12] and the World Health Organization [13] strongly recommend that, in order to gain competence and proper practice within the area of midwifery, clinical placements need to be supervised by a designated preceptor. It is important that a preceptor has all the right attributes that can favor the student in becoming engaged during practical training [14].

The aim of this study is to create and validate a tool designated to evaluate the attributes of a midwifery preceptor.

In the Italian context, there are no validated tools available to evaluate these attributes. The creation of a questionnaire form for students aims to identify and measure the right attributes of a preceptor, underlining and evaluating the responsibilities of future health professionals [15].

## 3. Methods

The aim of this study is twofold. Firstly, it wants to create a tool to be used by midwifery students in order to assess their preceptors, and secondly it aims to validate the tool so created. In order to achieve these goals, seven steps have been followed.

To achieve the first aim, the first three steps have been used; first, a literature review of the existing works concerning evaluation tools for midwifery preceptors has been done. The second step was to organize a focus group of midwifery students, in order to confirm or change the attributes tested in a preceptor previously identified through step number one, and to identify underlying items of different attribute areas. Step number three was to submit these areas and items identified by the students to a group of expert midwives via a meeting.

### 3.1. Step Number One: Literature Review

This research was carried out searching the databases of the National Library of Medicine (MEDLINE), with interface PubMed, and the Cumulative Index of Nursing and Allied Health Literature (CINHAL), setting a time limit of 10 years. The search string was: midwif*[ti] OR midwiv*[ti] OR midwifery AND mentors OR preceptor ship OR preceptor*[ti] OR bachelor*[ti] OR mentorship[ti]. Results obtained were then analysed and triangulated by researchers who were able to identify areas of preceptor attributes.

### 3.2. Step Number Two: Focus Group

Midwifery students from the Lombardy region were invited to participate in a focus group. They were chosen by purposive sampling, considered as experts by those students who had had at the end of their second and third year a clinical placement evaluation score ≥28/30 (in Italy 30 is the highest mark). The focus group was carried out after classes, at university, paying attention to confidentiality. This made use of a questio’ grid (Table 1). Conversations were recorded and transcribed. Anonymity for participants was guaranteed using pseudonyms. The transcript analysis was made by three researchers using Kanizsa’s method [16]: this sets data saturation when there is a repetition of identical/similar words, meanings, or expressions. Results obtained with data saturation were defined by three researchers and then triangulated with participants in the focus group [17].

### 3.3. Step Number Three: Meeting between Expert Midwives

A group of expert midwives was selected, through purposive sampling for the meeting. They all had ten years of experience in at least one of these fields: teaching, preceptorship, clinical management, practice development.

### 3.4. Step Number Four, Five, Six and Seven: Compilations by Students and Experienced Midwives

The remaining four steps of the study were carried out to achieve the second aim of the study which was to scientifically validate the results created. Step number four was to ask midwifery students at the University of Milan, during the academic year 2018–2019, to fill in the form, in order to select preceptors with a numeric score ≥4 so that ten preceptors each receive four valuation papers, filled in by different students.

To proceed to step five, similar items (spy items) were put in each form.

Step number six consisted of asking the students to assess their global experience with the preceptor on a visual analogue scale (VAS) at the end of the form.

The last step, number seven, was to ask expert midwives to fill in the forms of the preceptors, already assessed by students who had obtained a numeric score ≥4 four times.

### 3.5. Measures

Students were asked about some personal data: gender, age, year of the course. Expert midwives were asked about: gender, age, years of teaching, preceptorship and/or clinical management. To measure the items on the assessment form, a 4-point Likert scale was used. The VAS was drawn with a line 10 cm long: on its left extremity, there was the w0rding “not satisfactory”, and on its right, the wording “very satisfying”. Values were calculated measuring the distance in centimeters from the marked point to “not satisfactory”.

An Excel database was created to transcribe all the values from the Likert scale and VAS.

### 3.6. Statistical Analysis

Statistical analysis was carried out using software SAS ver. 9.2 (SAS Institute Inc., Carry, NC, USA). Consistency between items was measured with Cronbach Alpha for forms filled in by students and by expert midwives. Cronbach Alpha is based on the ratio between variability of single scores and variability of their sum. Its values are >0.90 excellent; between 0.80–0.90 good; between 0.70–0.79 fairly good; between 0.60–0.69 satisfactory; <0.60 inadequate.

Correlation between normalized values and VAS of each form (both students’ and midwives’) were figured with a scatter plot and measured with Pearson Correlation Coefficient (*r*).

### 3.7. Ethical Considerations

The study was carried out under the Declaration of Helsinki (last review: Seoul October 2008).

The Ethics Committee approved the study (No. 57/13), and written consent was obtained from all participants. Data was treated confidentially. Both student and midwife feedback were reported without the identity of either being disclosed.

This research did not receive any specific grant from funding agencies in the public, commercial, or not-for-profit sectors.

## 4. Results

### 4.1. Creation of Clinical Preceptors’ Assessment Tool

Step number one allowed the selection of six articles: three qualitative studies [4,5,6], two grounded theories [3,9], and one review [7]. These six studies allowed the summarization of four areas of competence for a preceptor: professional skills, interpersonal skills, pedagogical skills, personal qualities. This analysis was confirmed by results from the focus group.

Ten focus groups took place in four Universities within the Lombardy region: four in the University of Milan, two in the University of Milan Bicocca, two in the University of Brescia, two in the University of Pavia. For each university there was the same number of focus groups, for students of the second and third year of their course. Each focus group had from 7 to 15 Italian students, making a total of 88 students. Characteristics of students who took part in focus groups are given in Table 2. Each focus group lasted from 44 to 63 min (average 52 min) and they were conducted by a researcher. They confirmed the four areas of competence previously individuated through the literature review, and also 31 individual items gained by data saturation [18], obtained using Kanizsa’s method [16].

The four areas and 31 items identified were analyzed in a meeting between expert midwives (step number three). The meeting involved eight expert midwives, whose characteristics are reported in Table 2. This took 96 min and was conducted by two researchers who were allowed to draw up the definitive format of the preceptor’s assessment form. The ultimate form is made up of four areas of attitude and 33 items of competence, because the expert midwives decided that items n. 10, 24 and 28 had to be similar in order to evaluate the internal coherence of the form (spy items). The expert midwives established that each item had to be expressed with a positive value and a Likert score from 1 to 4. Score 1 was if the preceptor had “very little” for that characteristic, score 2 for “little”, score 3 if the preceptor had “much” for that characteristic, and score 4 for “very much”. The midwifery preceptor’s assessment form is shown in Table 3.

### 4.2. Validation of Clinical Preceptors’ Assessment Tool

For the academic year 2018–2019, 360 preceptor’s assessment forms were filled in and 10 preceptors were evaluated ≥4 times, for a total of 47 forms filled in by students (step number four). Each of these 10 preceptors was also evaluated by five of the eight expert midwives (Table 3), for a total of 50 forms filled in by senior midwives (step number seven).

Cronbach alpha calculated on forms filled in both by students and expert midwives (with specific scores for spy items) obtained excellent and good values; only rarely were they fairly good in a way that was superimposable between those filled in by students and those by expert midwives. (Table 4).

**Figure 1:**
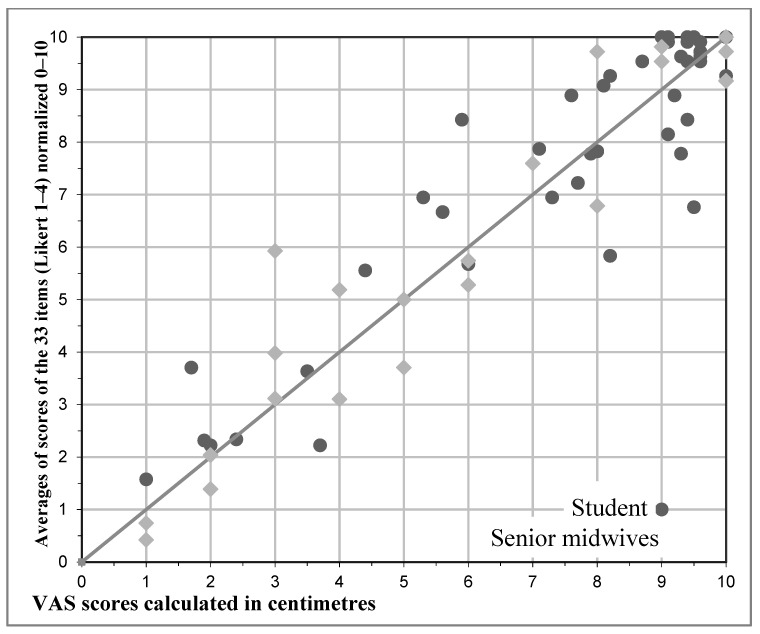
Pearson Correlation Coefficients: for the forms of the 10 preceptors who ≥4 evaluations from students, a comparison between the averages of scores of the 33 items (Likert 1–4), normalized 0–10 and VAS scores given by students and senior midwives calculated in centimeters was made, obtaining a Pearson Correlation Coefficient *r* = 0.78.

Lastly for the forms of the 10 preceptors who obtained ≥4 evaluations from students, a comparison between the averages of scores of the 33 items (Likert 1–4) normalized 0–10 and VAS scores calculated in centimeters was made, obtaining a Pearson Correlation Coefficient *r* = 0.78 (Figure 1).

## 5. Discussion

This study had two aims: to create a midwifery preceptor’s assessment form and to validate it: both were realized. The form showed a high degree of internal coherence and identified four areas of competence for a midwifery preceptor in order to be considered very good at clinical tutorship.

The first area disclosed as being a relevant quality for a preceptor is professional skill [6,7]. Preceptors have to keep their competences up to date [13,19,20] so that students do not perceive discrepancies between theoretical notions and clinical practice [21]. In the event that clinical plans are different from best practice, a preceptor must be able to justify this [22,23]. The focus group confirmed how important it is for students to be placed side by side with a preceptor, who is a professional model to be inspired by. and who works following clinical practice indicated by the Midwifery University Course [1,19,24].

The second area of competence disclosed by the focus group and the meeting between expert midwives underlines how effective communication and empathy in a preceptor positively influence the clinical placement experience of a student [7,9]. A preceptor must be helpful in both listening to a student’s questions while encouraging him to ask questions, [5] and using accurate communication when answering [7]. In addition, a student must feel himself/herself assessed on their expected skills (training objectives), not on personality traits [6,25].

The pedagogical skills area highlighted behaviour that can facilitate learning: to instill confidence in students’ capabilities so that they can fulfil their potential [6,11,25]; to provide opportunities for learning based on year of course [19]; and to supervise a student in order to gradually make them achieve more autonomy [23,26]. The literature, the students and senior midwives all consider it very important that a preceptor at the beginning of clinical placement shows the student how a ward is organized so that the undergraduate can settle in more easily [7]. A preceptor must know well what the goals are that a student must achieve in line with their University Course. [19,26] Furthermore the preceptor has to work to make the student reach them, in order to plan their learning in an effective way [7].

To validate the midwifery preceptor’s assessment form, two measures were used. First Cronbach’s alpha was used to measure item consistency, as to date it has been used in similar studies [27] despite its well-known limitations [27]. To compare scores submitted by students and senior midwives, the Pearson correlation coefficient was used, on the grounds that it is typically used for jointly/normally distributed data (data that follows a bivariate normal distribution) [28,29].

## 6. Conclusions

This study confirmed what the literature has highlighted: that it is very important to provide constructive comments and feedback to students during and after their clinical placements [5,7,14,27,30]. Feedback can be given at any time during placements, provided that privacy is maintained and that professional development is promoted [3,25].

The last area of attributes and its five items identified the personal qualities a preceptor should have. Preceptors must be able to recognize their limits [5]; have passion for the profession and show enthusiasm in sharing their knowledge with students [4,28,31]. The professional model embodied by a preceptor is crucial, as a student will try to emulate it [5,29,32].

The midwifery preceptor’s assessment form created via this study was confirmed in all parts by the expert midwives, and this was shared with students’ experiences.

The achievement of the creation of this midwifery preceptor’s assessment form fills a gap in the evaluation system of Italian Midwifery Courses and will be able to level the requirements of a preceptor [30,31,32,33]. Researchers hope these requirements will become the focus of courses or Master’s degrees for midwives who intend to study to become qualified preceptors.

## Figures and Tables

**Table 1:** Focus group grid.

1. What do you think a preceptor should be like?
2. Literature indicates a preceptor must have professional skills, interpersonal skills, pedagogical skills and personal qualities. Do you agree with these four categories of attributes?
3. Would you add or remove any of the categories of attributes?
4. What skills do you think a preceptor should have for each area of competence that has been identified?

**Table 2:** Characteristics of the participants of focus groups [student *n* = 88] and of meeting between senior midwives [expert midwife *n* = 8].

**Variables**	**Participants Focus Group (N = 88)**	
	**N**	**%**
Gender		
F	87	98.9
M	1	1.1
Age		
20–21	33	37.5
22–23	30	34.1
23–24	17	19.3
25–26	4	4.5
27–28	2	2.3
>29	2	2.3
Year of course		
Second	42	47.7
Third	46	52.3
**Variables**	**Participants meeting (N = 8)**	
	**N**	**%**
Gender		
F	8	100.0
Age (years)		
40–44	1	12.5
45–49	3	37.5
50–54	2	25.0
55–59	2	25.0
Experience (years)		
Teaching		
No teaching	4	50.0
10	2	25.0
11–20	1	12.5
≥21	1	12.5
Preceptorship		
No preceptorship	0	00.0
10	2	25.0
11–20	2	25.0
≥21	4	50.0
Clinical Management		
No clinical management	3	37.5
10	1	12.5
11–20	2	25.0
≥21	2	25.0

**Table 3:** Midwifery preceptors’ assessment tool.

Tick your year of course: □ I □ II □ III	Score
Dear Student, We Ask you to Give a Score for each of the 33 items regarding the preceptor you have been assigned to (Name and Surname of the preceptor) _________________________________	
Scores for items: 4= very much 3 = much 2 = little 1 = very little	
1. PROFESSIONAL SKILLS	
1. Is able to provide explanations	
2. Makes use of scientific evidence during clinical practice	
3. Integrates theory and practice	
4. Provides explanations when clinical practice deviates from scientific evidence and/or protocols	
2. INTERPERSONAL SKILLS	
5. Has good communicative skills	
6. Is respectful	
7. Makes you comfortable	
8. Considers you as a member of the team	
9. Encourages you to ask questions	
10. Gives you feedback during practice	
11. Assess you on a professional level, not on a personal one	
3. PEDAGOGICAL SKILLS	
12. Shows you how the ward is organized at the beginning of your clinical placement	
13. Knows what learning objectives are	
14. Compares with you on your expectations and your learning objectives	
15. Puts you at the center of the tutorial relationship	
16. Values your past knowledge and experience	
17. Provides you with learning opportunities	
18. Helps you to believe in your potential	
19. Spurs you to autonomy	
20. Supervises you during your new experiences without interfering	
21. Facilitates your awareness in professional responsibility	
22. Helps your critical thinking	
23. Appreciates activities you perform correctly	
24. Is objective in providing feedback	
25. Helps you in debriefing	
26. Helps you in having a global vision of clinical situations	
27. Accepts your mistakes and corrects them in the most helpful way	
28. Compares with you and shares the valuation during placement and once it is ended	
4. PERSONAL QUALITIES	
29. Is punctual when a shift starts	
30. Admits his/her own limits	
31. Shows passion for his/her job	
32. Shows enthusiasm in teaching	
33. Is a professional model that inspires you	
GLOBAL JUDGMENT	
In the box below, tick with a X the point which corresponds to the judgment you give to your preceptor	
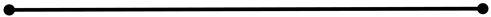	

**Table 4:** Cronbach Coefficient Alpha calculated on forms filled in by students (*n*. 47) and expert midwives [*n*. 50].

Cronbach Coefficient Alpha with Deleted Variable								
	Student				Expert Midwives			
Deleted Variable	Raw Variables		Standardized Variables		Raw Variables		Standardized Variables	
	Correlation with Total	Alpha	Correlation with Total	Alpha	Correlation with Total	Alpha	Correlation with Total	Alpha
Professional skills	0.82	0.93	0.83	0.93	0.94	0.96	0.93	0.96
Interpersonal skills	0.85	0.92	0.84	0.92	0.96	0.96	0.96	0.95
Pedagogical skills	0.90	0.90	0.90	0.90	0.95	0.96	0.94	0.96
Personal qualities	0.85	0.92	0.85	0.92	0.90	0.97	0.89	0.97
Item 10	0.78	0.71	0.79	0.72	0.95	0.95	0.95	0.95
Item 24	0.71	0.79	0.71	0.79	0.93	0.97	0.93	0.97
Item 28	0.65	0.84	0.65	0.85	0.95	0.95	0.95	0.95
